# Global miRNA expression reveals novel nuclear and mitochondrial interactions in Type 1 diabetes mellitus

**DOI:** 10.3389/fendo.2022.1033809

**Published:** 2022-11-24

**Authors:** Rafaella Sousa Ferraz, Lucas Cauê Bezerra Santos, Rebecca Lais da-Silva-Cruz, Cintia Helena Braga-da-Silva, Leandro Magalhães, Arthur Ribeiro-dos-Santos, Amanda Vidal, Tatiana Vinasco-Sandoval, Laís Reis-das-Mercês, Camille Sena-dos-Santos, Adenilson Leão Pereira, Lilian Souza D’Albuquerque Silva, Franciane T. Cunha de Melo, Ana Carolina C. Braga de Souza, Valéria S. Galvão Leal, Priscila B. Barbosa de Figueiredo, João F. Abrahão Neto, Lorena Vilhena de Moraes, Gabriela Nascimento de Lemos, Natércia Neves Marques de Queiroz, Karem Miléo Felício, Giovanna C. Cavalcante, Ândrea Ribeiro-dos-Santos, João Soares Felício

**Affiliations:** ^1^ Laboratory of Human and Medical Genetics, Graduate Program in Genetics and Molecular Biology, Federal University of Para, Belem, PA, Brazil; ^2^ Instituto Tecnológico Vale Desenvolvimento Sustentável Vale, Institute of Technology, Belem, PA, Brazil; ^3^ Laboratoire de Génomique et Radiobiologie de la Kératinopoïèse, Institut de Biologie François Jacob, CEA/DRF/IRCM, Evry, France; ^4^ Medical School, Federal University of Para, Altamira, PA, Brazil; ^5^ Endocrinology Research Center, Joao de Barros Barreto University Hospital, Federal University of Para, Belem, PA, Brazil; ^6^ Oncology Research Center, Graduate Program in Oncology and Medical Sciences, Joao de Barros Barreto University Hospital, Federal University of Para, Belem, PA, Brazil

**Keywords:** type 1 diabetes, miRNAs, miRnome, nuclear target, mitochondrial target

## Abstract

**Background:**

Considering the potential role of miRNAs as biomarkers and their interaction with both nuclear and mitochondrial genes, we investigated the miRNA expression profile in type 1 diabetes (T1DM) patients, including the pathways in which they are involved considering both nuclear and mitochondrial functions.

**Methods:**

We analyzed samples of T1DM patients and control individuals (normal glucose tolerance) by high throughput miRNA sequencing (miRNome). Next, five miRNAs – *hsa-miR-26b-5p*, *hsa-let-7i-5p*, *hsa-miR-143-3p*, *hsa-miR-501-3p* and *hsa-miR-100-5p –* were validated by RT-qPCR. The identification of target genes was extracted from miRTarBase and mitoXplorer database. We also performed receiver operating characteristic (ROC) curves and miRNAs that had an AUC > 0.85 were considered potential biomarkers.

**Results:**

Overall, 41 miRNAs were differentially expressed in T1DM patients compared to control. *Hsa-miR-21-5p* had the highest number of predicted target genes and was associated with several pathways, including insulin signaling and apoptosis. 34.1% (14/41) of the differentially expressed miRNAs also targeted mitochondrial genes, and 80.5% (33/41) of them targeted nuclear genes involved in the mitochondrial metabolism. All five validated miRNAs were upregulated in T1DM. Among them, *hsa-miR-26b-5p* showed AUC>0.85, being suggested as potential biomarker to T1DM.

**Conclusion:**

Our results demonstrated 41 DE miRNAs that had a great accuracy in discriminating T1DM and control group. Furthermore, we demonstrate the influence of these miRNAs on numerous metabolic pathways, including mitochondrial metabolism. *Hsa-miR-26b-5p* and *hsa-miR-21-5p* were highlighted in our results, possibly acting on nuclear and mitochondrial dysfunction and, subsequently, T1DM dysregulation.

## 1 Introduction

Type 1 diabetes mellitus (T1DM) is an autoimmune disease associated with failure in insulin production that occurs as a consequence of the pancreatic islet β-cells dysregulation mediated by T-cells (1). This type of Diabetes Mellitus (DM) can affect any age group, but the onset is more frequent in children and adolescents (2). Globally, 1.1 million individuals under the age of 20 years are affected by T1DM, with an annual increase of about 3% (3).

T1DM is an immune-based disease driven by the interaction between environmental, genetic, and epigenetic factors (4, 5). The presence of autoantibodies is the first sign of autoimmunity against β-cells (6). Currently, the standard method to identify individuals at risk for T1DM is to analyze the presence of autoantibodies against islet antigens, among them, against islet cells (ICA), glutamate decarboxylase (GADA), insulin (IAA), tyrosine phosphatases (IA-2 and IA-2β), and zinc transporter 8 (ZnT8) (7). Antibodies are the most common biomarkers of T1DM, but only a portion of the autoantibody-positive individuals develop the disease. Thus, new biomarkers are required to help the identification of T1DM patients (8).

Several microRNAs (miRNAs) – short non-coding RNAs (~22 nucleotides) that play important roles in the gene expression regulation (9) – have been reported in association with T1DM, affecting β-cell metabolism (10, 11), insulin secretion (12, 13), T-cell function (14, 15), biosynthesis and performance of autoantigens (16). Furthermore, it has been demonstrated that the miRNAs act on T1DM not only *via* nuclear, but also *via* mitochondrial pathways. The miRNA-181a, for example, is overexpressed in T1DM patients compared to control and it is able to inhibit hydrogen peroxide-induced cellular apoptosis, lead to disruption of mitochondrial structure, increase ROS (reactive oxygen species) production, and downregulate the expression of mitochondrial anti-apoptotic proteins (10, 17).

In this context, we explored miRNA expression profiles in T1DM patients through miRNome sequencing and investigated the pathways these miRNAs are involved considering both nuclear and mitochondrial functions.

## 2 Material and methods

### 2.1 Ethics statement

This study was approved by the Institutional Review Board from João de Barros Barreto University Hospital (HUJBB, Belém, Pará, Brazil) (Protocol Number 005/12). All procedures performed involving human participants were conducted according to the ethical guidelines of the Declaration of Helsinki. Written informed consent was obtained from all study participants.

### 2.2 Sample collection

**Figure 1:**
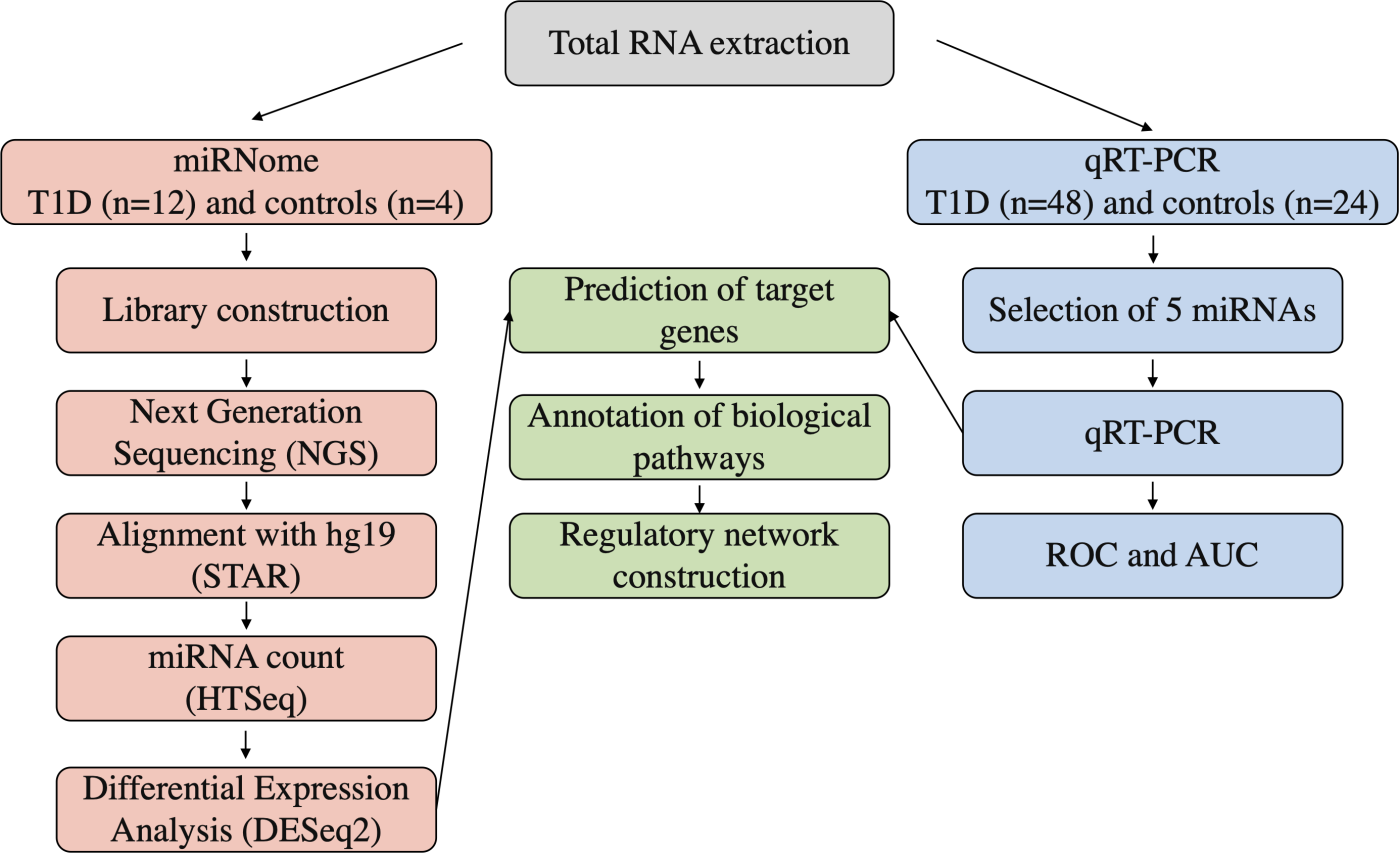
Experimental workflow of the analyses in the study.

Sixty patients with T1DM – diagnosed according to the American Diabetes Association (ADA) criteria (18) – and twenty-eight subjects with normal glucose tolerance (control individuals) were enrolled in the current study by the Endocrinology and Metabology/Diabetes Unit at HUJBB. T1DM group had mean age 26.93 ± 9.62 years, with individuals equally distributed between females and males. The mean age of the control group was 28.83 ± 6.85 years old, with predominance of females (75%). Peripheral blood samples were collected into a Tempus Blood RNA tube (Thermo Fisher Scientific, Waltham, MA, USA) and stored at -20°C until RNA extraction. A summary of our experimental workflow is presented in **Figure 1**.

### 2.3 Total RNA isolation and quantification

Total RNA was extracted using MagMAX™ RNA Isolation Kit (Thermo Fisher Scientific, Waltham, MA, USA) according to manufacturer’s specification and quantified with NanoDrop-1000 spectrophotometer (Thermo Fisher Scientific, Waltham, MA, USA). The RNA integrity was determined using Agilent RNA ScreenTape assay and 2200 TapeStation Instrument (Agilent Technologies, Santa Clara, CA, USA).

### 2.4 Library preparation and next-generation sequencing

A high throughput small RNA-sequencing experiment was conducted in 12 patients with T1DM and 4 control individuals. For library preparation, 1 µg of total RNA per sample was used with TruSeq Small RNA Library Preparation (Illumina, San Diego, CA, USA). The library was validated and quantified by DNA ScreenTape assay in a 2200 TapeStation Instrument (Agilent Technologies, USA) and by real-time PCR with a KAPA Library Quantification Kit (Roche, Basel, Switzerland). A total library pool of 4 nM was sequenced using a MiSeq Reagent Kit v3 (150 cycles) at the MiSeq System (Illumina).

### 2.5 Sequencing data processing and differential expression analysis

A pipeline of quality control to remove adapters and filter low quality reads was applied using Trimmomatic software (19). Resulting sequences were aligned with the human genome reference (Hg19) using STAR software (20). Mature miRNAs sequencing was quantified using miRbase human annotation and expression count was performed with HTSeq software (21).

The DE analysis was performed using the Bioconductor-DESeq2 package (22) in R software. Comparisons between patients with T1DM and control individuals were conducted. Adjusted values of *p* ≤ 0.05 and a |log2 fold change| ≥ 1.5 were considered statistically significant. Graphical analysis of miRNAs was normalized to RPKM (Reads per Kilobase per Million). Heatmap was used for hierarchical clustering of DE miRNAs.

### 2.6 Validation by quantitative real-time reverse transcription-PCR

Based on the NGS data, five miRNAs – *hsa-miR-26b-5p*, *hsa-let-7i-5p*, *hsa-miR-143-3p*, *hsa-miR-501-3p* and *hsa-miR-100-5p* – were selected for validation by Real‐Time Quantitative Reverse Transcription‐PCR (RT‐qPCR). The experiment was conducted in 48 patients with T1DM and 24 control individuals. Total RNA was used in a reverse transcription reaction using miRNA 1st Strand cDNA Synthesis Kit (Agilent Technologies). The reverse transcription product was subject to amplification using PowerUp SYBR Green Master Mix in ABI 7500 Real Time PCR System (Thermo Fisher Scientific). The specific primers for mature miRNAs are listed in **Supplement Table S1**. All reactions were performed in triplicate, and the comparative Ct method was used to analyze the differences in the expression of each group. The expression levels of miRNAs were normalized by using the endogenous control small nucleolar RNA U6.

### 2.7 ROC and AUC analyses

To estimate the biomarker sensitivity for distinguishing groups, Receiver Operating Characteristics (ROC) and Area Under the Curve (AUC) analyses were used. These measures are effective in discriminating the true state of individuals, being a standard analysis for searching for biomarkers. In this study, the miRNAs that demonstrated AUC>0.85 were considered potentially useful for the diagnosis of T1DM.

### 2.8 Identification of target genes and functional enrichment analysis

Target genes of DE miRNAs were extracted from miRTarBase database (access in July 2021) (23) considering only those that were validated by strong evidence (report assay, western blot, and qPCR). Enrichment analysis of the target genes were conducted in both KEGG and Reactome pathways using the ReactomePA (24) and ClusterProfiler package (25) in R. Enriched terms with an FDR adjusted p-value < 0.05 were considered statistically significant. Interaction network of miRNA-target gene and target gene-biological pathways were constructed using Cytoscape (26). For the mitochondrial approach, we based our analyses on the human mitochondrial interactome (mitochondrial functions and their associated genes) present in the mitoXplorer platform (27).

### 2.9 Statistical analyses

Statistical analyses and graphing were performed using R software (28). Shapiro-Wilk test was used to evaluate the data distribution. To evaluate the statistical significance between the analyzed groups, we used Mann-Whitney U test. P value < 0.05 was considered statistically significant.

## 3 Results

### 3.1 Identification of differentially expressed miRNAs in type 1 diabetes

**Figure 2:**
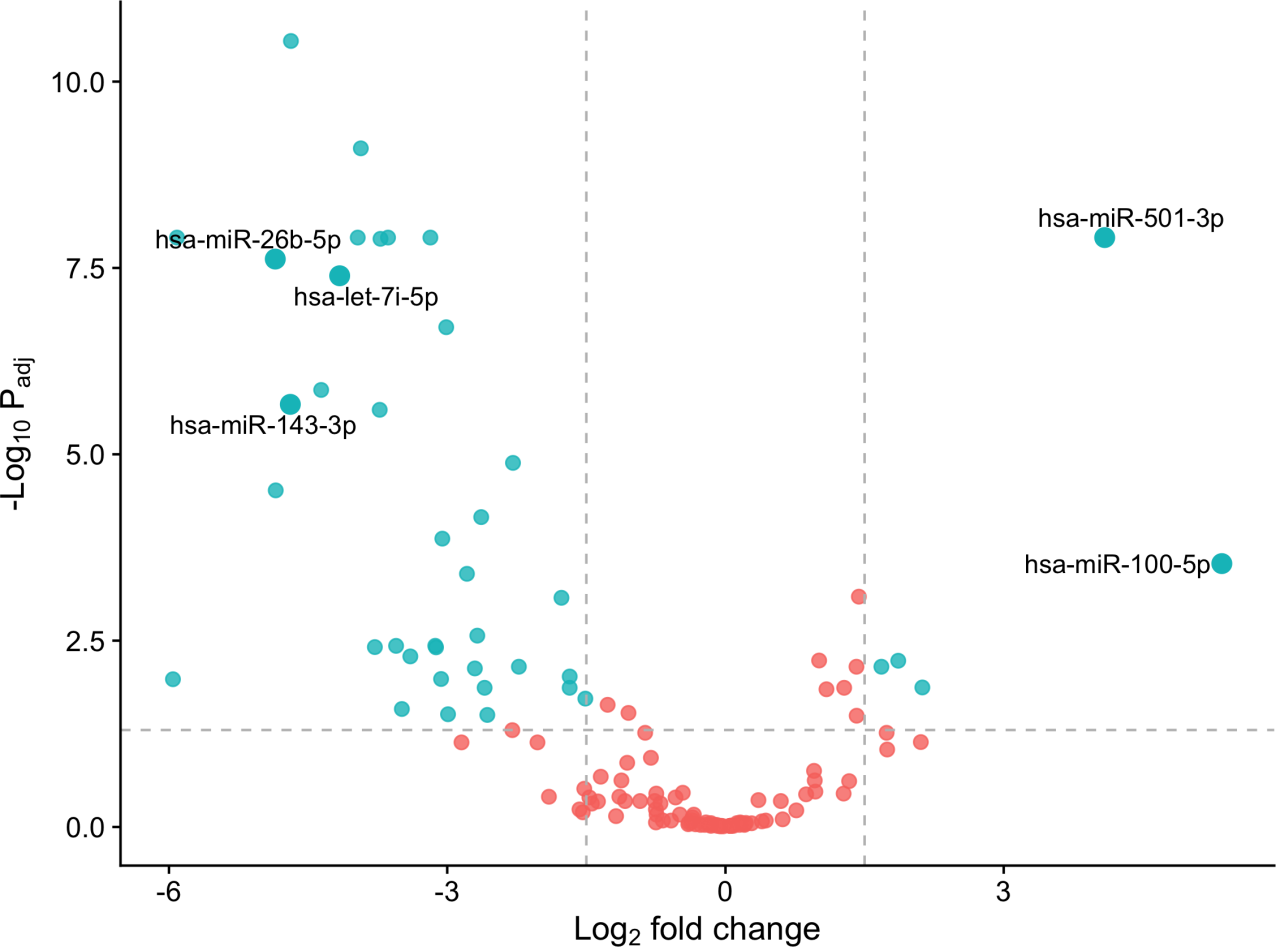
DE miRNAs in the T1DM patients in comparison to controls. Blue dots are considered DE miRNAs under the conditions of adjusted values of *p* < 0.05 and |log2 fold change| ≥ 1.5. Red dots are non-DE miRNAs. Note that miRNAs on right of figure are up-regulated, and on the left are down-regulated.

**Figure 3:**
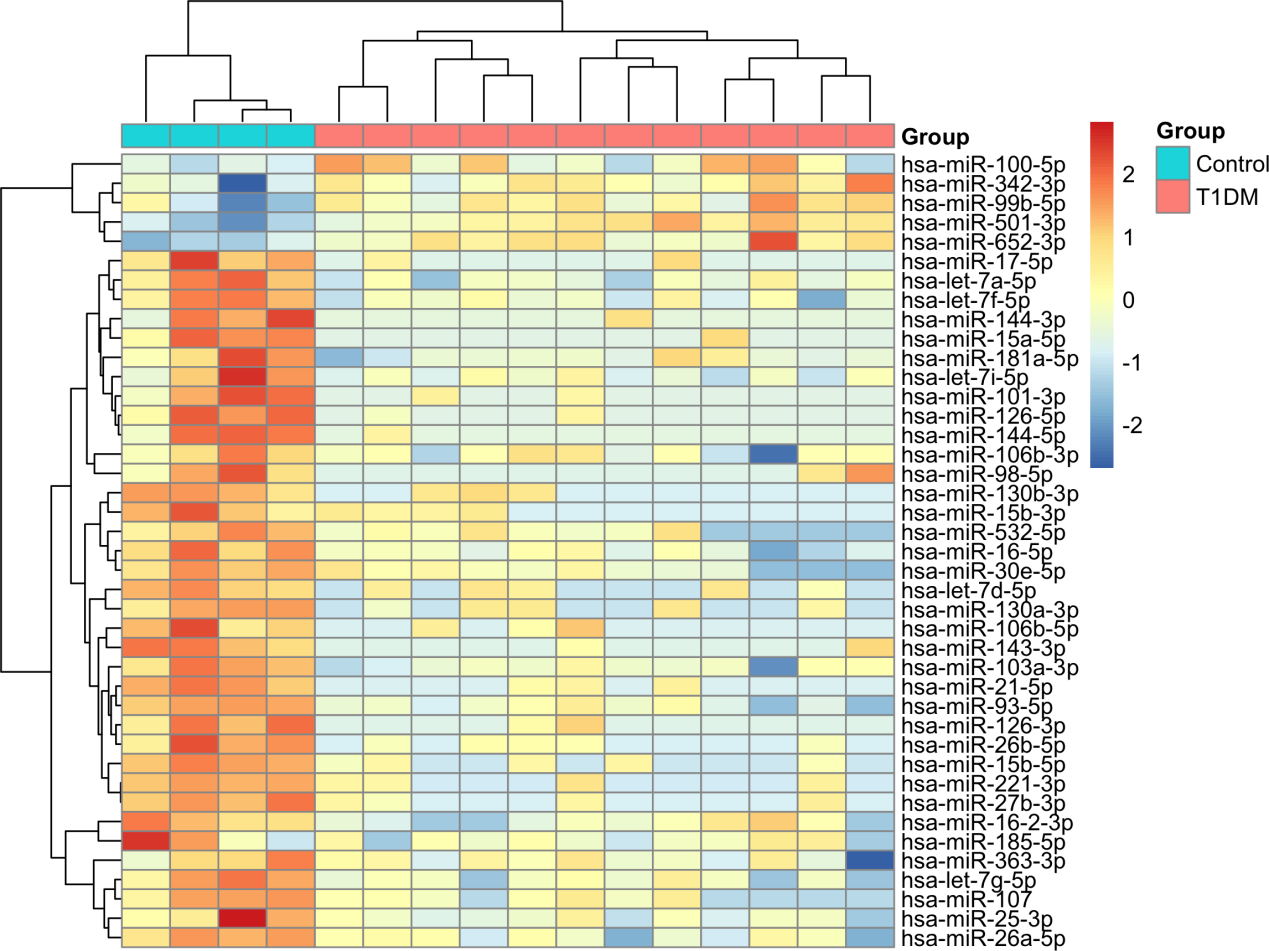
Heatmap with hierarchical clustering analysis of DE miRNAs in T1DM. Blue color in top bar represents control individuals and red colors represents T1DM patients. In the heatmap, dark-red color corresponds to high miRNA expression, and dark blue corresponds to low expression.

We identified 41 differentially expressed (DE) miRNAs in patients with T1DM in comparison to control individuals, including 36 downregulated and 5 upregulated miRNAs (**Figure 2**). Hierarchical clustering of normalized expression of these miRNAs provided a heatmap graph that clearly separated T1DM and control group (**Figure 3**).

### 3.2 Target genes identification

**Figure 4:**
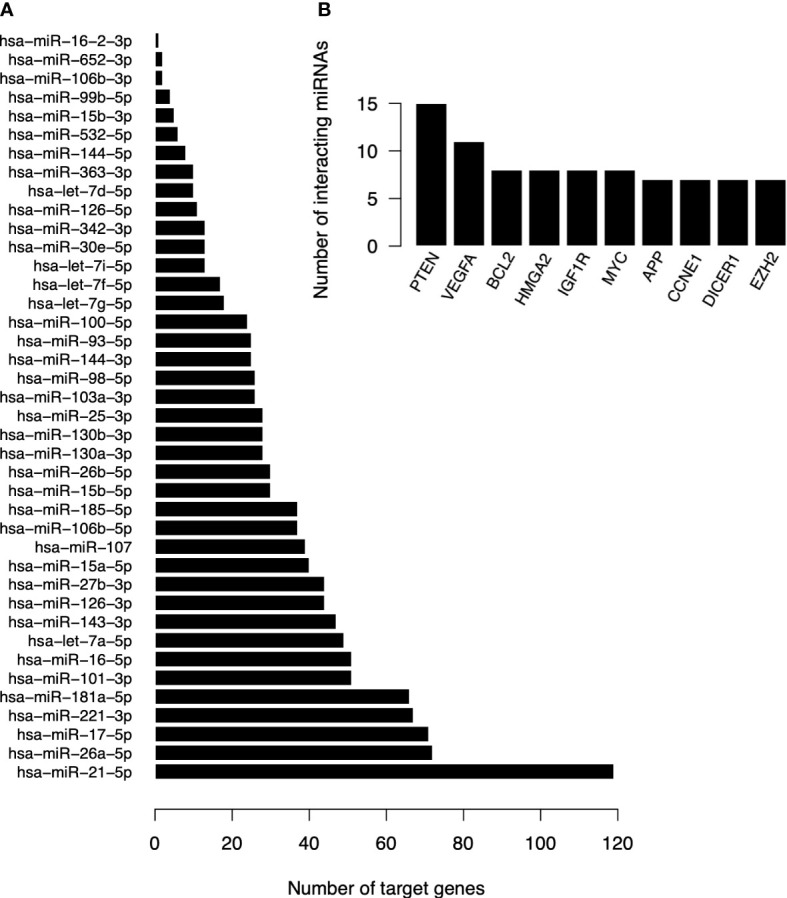
Quantitative of target genes of DE miRNAs in T1DM. **(A)** Number of target genes per DE miRNA. **(B)** Number of interacting miRNAs for the ten most frequently found genes.

We identified 1,237 interactions with 777 target genes (**Supplement Table S2**). Only *hsa-miR-501-3p* had no predicted target genes with strong evidence. The *hsa-miR-21-5p* was by far the miRNA with the highest number of targets (**Figure 4A**). On the other hand, *PTEN* and *VEGFA* were the genes with the greater number of interactions with different miRNAs, followed closely by *BCL2*, *HMGA2*, *IGF1R* and *MYC* (**Figure 4B**).

### 3.3 Validation of miRNAs expression by RT-qPCR

**Figure 5:**
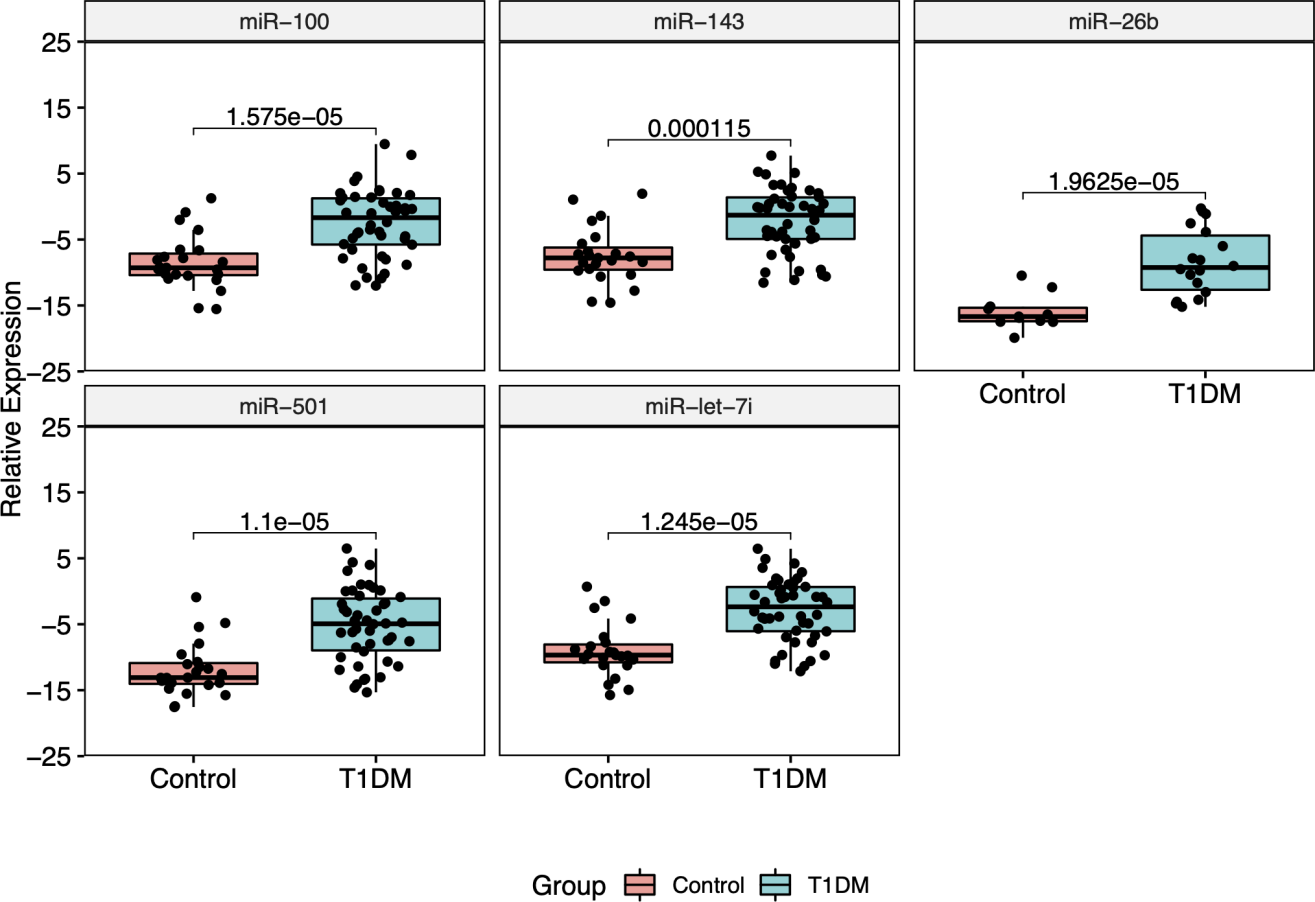
Relative expression of five RT-qPCR validated miRNAs. All of them showed to be upregulated in T1DM group.

**Figure 6:**
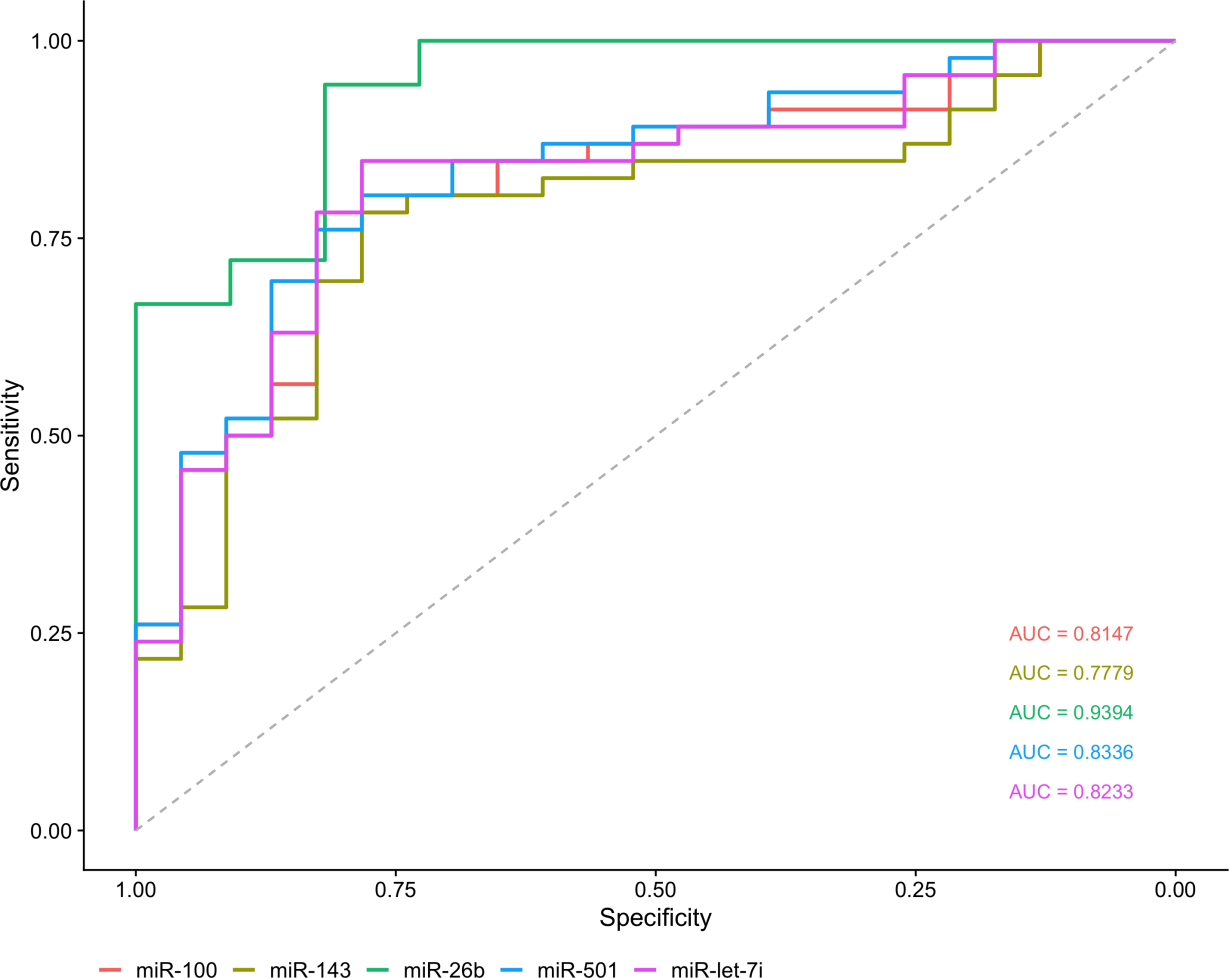
Analysis of biomarker sensitivity of five validated miRNAs in T1DM.

The five most DE miRNAs, considering the lowest p-value and highest log_2_ fold change, were selected to be validated by RT-qPCR: *hsa-miR-100-5p*, *hsa-miR-501-3p, hsa-miR-143-3p, hsa-let-7i-5p* and *hsa-miR-26b-5p.* All of them were upregulated in T1DM in comparison to control (**Figure 5**). Only *hsa-miR-26b-5p* showed AUC>0.85, being highlighted as a potential biomarker to T1DM (**Figure 6**).

**Figure 7:**
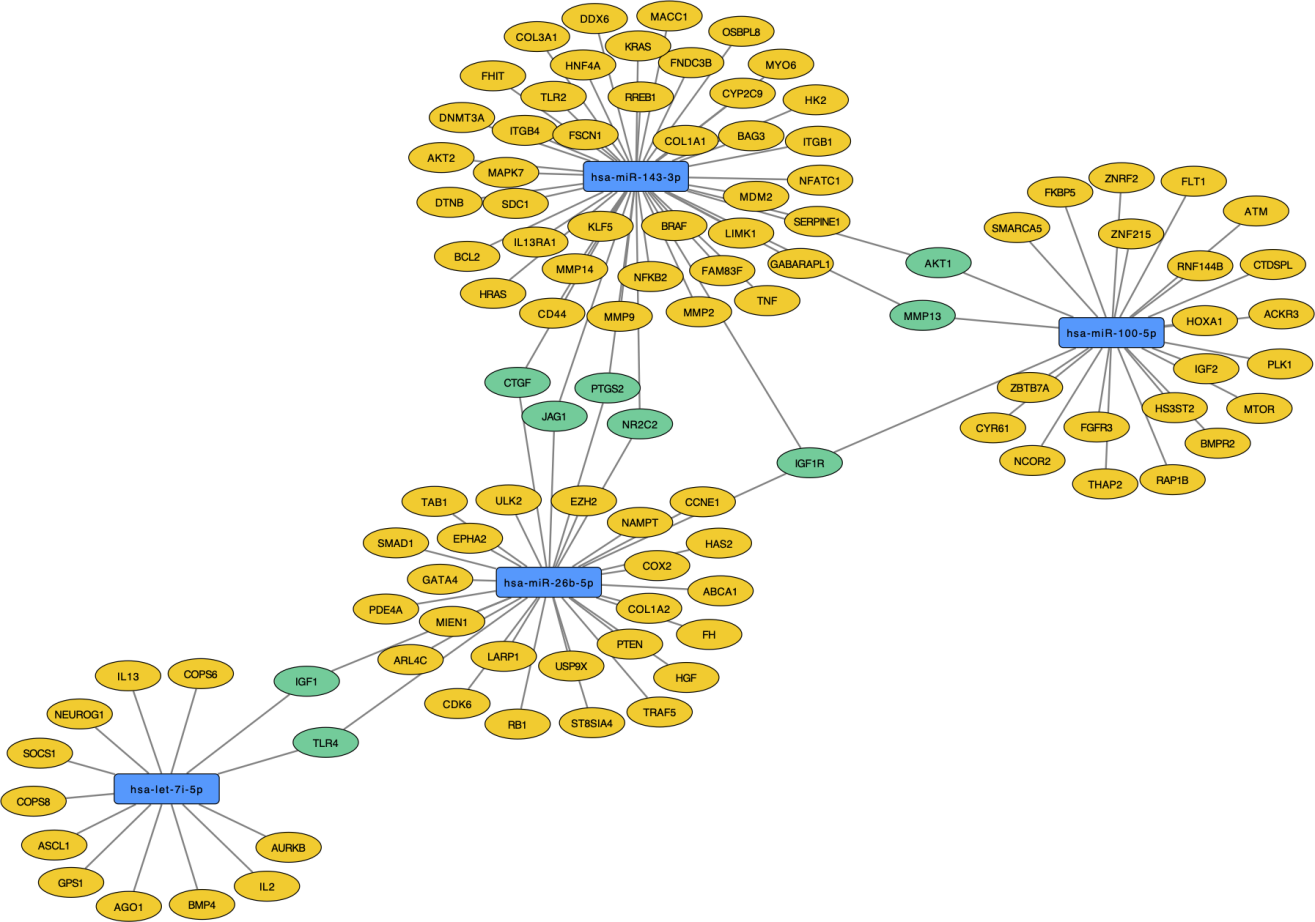
Target genes of validated miRNAs (blue) with strong evidence in T1DM. Those regulated by at least two of the miRNAs are pictured in green and those solely regulated by one of the validated miRNAs are picture in yellow.

Interestingly, hsa-miR-100-5p, hsa-miR-143-3p, hsa-let-7i-5p, and hsa-miR-26b-5p regulate the genes IGF1, TLR4, CTGF, JAG1, PTGS2, NR2C2, IGF1R, MMP13 and AKT1. Only the IGF1R gene (Insulin Like Growth Factor 1 Receptor) was regulated by three of these miRNAs (hsa-miR-100-5p, hsa-miR-143-3p and hsa-miR-26b-5p), making it a central gene in T1DM regulatory network. Curiously, hsa-miR-26b-5p regulate both IGF1R and IGF1, which are genes related to insulin signaling and apoptotic events (**Figure 7**). The hsa-miR-501-3p did not have target genes of strong evidence, so it was removed from the analyses.

### 3.4 Functional enrichment analysis

To investigate the biological pathways that these 41 DE miRNAs play in the development of T1DM, we separated analyzes in miRNAs targeting nuclear genes and miRNAs targeting mitochondrial genes. These results are showed in the next sections.

#### 3.4.1 Nuclear

To improve the interpretation of the results, we divided the functional enrichment of miRNAs for nuclear genes into genes regulated by downregulated miRNAs and those regulated by upregulated miRNAs.

The functional analysis of the upregulated miRNAs revealed that its target genes participate in 61 KEGG pathways (**Supplement Table S3**). Among these, we highlight 22 that are important for the development of T1DM (**Supplement Figure S1**), including apoptosis pathway and protein complex signaling (TGF-β, EGFR, Pi3K-Akt, HIF-1, TNF, mTOR, hippo, Notch etc.)

The investigation of the downregulated miRNAs presented interaction with 751 nuclear genes that are involved in 150 KEGG pathways (**Supplement Table S4**), of which at least 40 pathways are related to T1DM (**Supplement Figure S2**). In addition to pathways already associated with upregulated miRNAs, we found pathways associated with the immunological response and insulin signaling.

#### 3.4.2 Mitochondrial

To better explore mitochondrial association with T1DM, we divided the miRNAs in two groups: miRNA targeting mitochondrially-encoded genes and miRNAs targeting nuclear genes involved in mitochondrial metabolism (NucGenMito).

**Figure 8:**
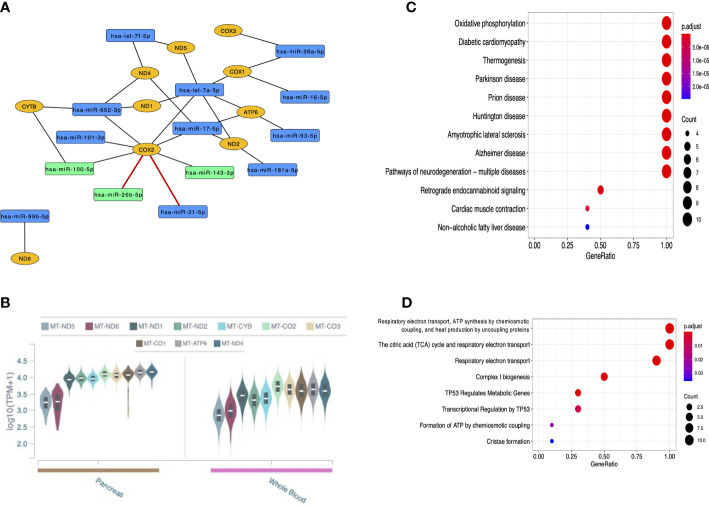
Analyses of the 14 DE miRNAs that target mitochondrially-encoded genes. **(A)** Network of these miRNAs (in blue or green, the latter being those validated in the current study) and the mitochondrial genes they target (in yellow); **(B)** GTEx data of mitochondrially-encoded gene expression in the pancreas and whole blood; **(C)** KEGG pathways of mitochondrial functions and some multifactorial conditions; **(D)** Reactome pathways of mitochondrial mechanisms.

In total, 14 miRNAs targeting mitochondrial genes were recognized. Curiously, eight of them were found interacting with *MT-COX2* (also known as *MT-CO2* and MT-*COII*; mitochondrially-encoded cytochrome c oxidase II) considering both strong and weak evidence interactions. Among these miRNAs are the three that were validated by RT-qPCR, although only the interactions with *hsa-miR-21-5p* and *hsa-miR-26b-5p* are of strong evidence in the global literature (in red) (**Figure 8A**). All mitochondrial genes targeted by miRNAs are expressed in the pancreas and whole blood from GTEx data (**Figure 8B**), reinforcing the potential role of these genes in T1DM. KEGG pathways and Reactome pathways are shown in **Figures 8C,D**, respectively, highlighting important mitochondrial mechanisms and some conditions that have been related to both nucleus and mitochondria.

Considering mitoXplorer database, 33 DE miRNAs targeting NucGenMito were reported, including those validated by RT-qPCR (**Supplement Figure S3A**). Most of these miRNAs are involved in Transcription (nuclear), Apoptosis and Mitochondrial Signaling (**Supplement Figure S3B**). *Hsa-miR-21-5p*, *hsa-miR-221-3p* and *hsa-miR-181a-5p* had the greater number of targets, over 10 target genes (**Supplement Figure S3C**). As 80.5% (33/41) of miRNAs targeting NucGenMito also targeted mitochondria-independent nuclear genes, functional analysis enriched for the same previously mentioned biological pathways (**Supplement Figures S4, S1-S2**).

## 4 Discussion

Circulating miRNAs are strong candidates to be biomarkers for complex diseases, including diabetes mellitus. In a way, because they are stable, resistant to ribonuclease, can be easily collected, and their level can be measured using assays that are rapid, specific, and sensitive (7). Therefore, we investigated the profile of miRNAs expressed in the blood from a cohort of T1DM patients, looking for potential new biomarkers for this disease.

Here, 41 miRNAs were found to be dysregulated in T1DM patients in comparison to controls, suggesting a potential role in T1DM development. Among these, 10 miRNAs (*hsa-miR-99b-5p*, *hsa-miR-501-3p*, *hsa-let-7f-5p*, *hsa-miR-143-3p*, *hsa-miR-144-3p*, *hsa-miR-181a-5p*, *hsa-miR-126-5p*, *hsa-miR-144-5p*, *hsa-miR-16-5p*, and *hsa-miR-25-3p*) are the same found in the previously whole-blood miRNA sequencing in diabetes performed by Massaro et al. (2019) (29), reinforcing the involvement of these miRNAs with the diabetes process. In the last-mentioned study, these miRNAs were also related to diabetes complications (*i.e.*, neuropathy, retinopathy, and nephropathy) and were able to differentiate T1DM patients from controls. Nonetheless, there is still limited literature on these miRNAs and T1DM currently.

In our study, it should be noted that *hsa-miR-21-5p* had the highest number of predicted target genes. This miRNA – together with others such as *hsa-miR-181a-5p* – has been reported in plasma/serum and Peripheral Blood Mononuclear Cells (PBMCs) acting as potential circulating biomarker in T1DM (6). In a recent study with breast cancer, *hsa-miR-21-5p* was reported to be sublocated in mitochondria and able to interact with mitochondria-related differentially expressed genes in multiple mechanisms (30), including the collagen metabolism by Discoiding Domain Receptor 2 (DDR2), which, in turn, has been related to diabetic osteopenia (31). Here, *hsa-miR-21-5p* was associated, among so many other pathways, with insulin resistance, apoptosis, and diabetic cardiomyopathy (**Supplement Tables S2, S4**). Curiously, these three pathways not only have been notably present in our findings but have also been associated to mitochondrial functions in T1DM in previous studies (32–34).

Moreover, we highlight the association of multiple miRNAs to *MT-COX2* in our study, particularly *hsa-miR-21-5p* and *hsa-miR-26b-5p* that were predicted with strong evidence. The *MT-COX2* gene encodes a subunit of the Complex IV (also known as cytochrome c oxidase), one of the five protein complexes in the electron transport chain (ETC) repeatedly located in the mitochondrial cristae and responsible for the energy generation during OXPHOS (35). Importantly, mitochondrial dysfunction leading to imbalanced OXPHOS activity has been reported in T1DM, including the decreased activity of ETC complexes in T1DM heart (36), although the specific mechanisms affected in these processes have not yet been clarified. In addition, it should be noted that oxidative stress by the accumulation of mitochondrial ROS – mainly due to hyperglycemia-induced mitochondrial dysfunction and altered dynamics and biogenesis – has been described as a key factor to T2DM and some of the diabetic complications, including insulin resistance (37, 38).

Curiously, insulin resistance has been related to serum levels of the growth factor IGF1 and its receptor IGF1R – components of the growth hormone (GH) and energy metabolisms (39, 40). In fact, the dysregulation of IGF1 and IGF1R levels has been described in association to hyperglycemia in diabetes, including T1DM, and several diabetic complications (40–42). In our study, *hsa-miR-26b-5p, hsa-let-7i-5p, hsa-miR-100-5p* and *hsa-miR-143-3p* were found to be interacting with *IGF1R* and/or *IGF1* gene, suggesting that these miRNAs might play a role in this insulin resistance metabolism, in addition to diabetic complications. Surprisingly, all three miRNAs (*hsa-miR-26b-5p, hsa-miR-100-5p* and *hsa-miR-143-3p*) were shown here to interact with *MT-COX2* gene, which is particularly relevant considering that IGFR1 and the GH/IGF1 axis have been related to mitochondrial function and dynamics (43, 44).

In addition, *hsa-miR-26b-5p* was recently described to form a signaling pathway with *Mfn1* (mitofusin 1), an essential gene for mitochondrial fusion; this *hsa-miR-26b-5p/Mfn1* axis seems to affect mitochondrial dynamics and apoptosis in the context of myocardial infarction and cardiac microvascular dysfunction (45). Therefore, *hsa-miR-26b-5p* could be especially important to diabetic cardiomyopathy and, possibly, other diabetic complications. To the best of our knowledge, this is the first study to highlight a potential key role of *hsa-miR-26b-5p* in T1DM development and progression.

Here, we describe the main regulatory dysfunctions in the miRNA pathways associated with T1DM, including their role at the nuclear and mitochondrial levels. To strengthen our results, we recommend future investigations on these miRNAs in cellular and animal models to validate the regulatory network in which they are involved. Of note, a major limitation of this study was the sample size, so we also recommend the validation of miRNAs in a larger cohort to guarantee the veracity of the results, in addition to patients with different diabetic complications. Despite these limitations, our findings contribute to the knowledge of complex regulation of T1DM and identification of miRNAs as potential biomarkers.

## Conclusion

In summary, we found differentially expressed miRNAs between T1DM patients and control individuals that clearly separate both groups. These miRNAs seem to regulate multiple nuclear and mitochondrially-encoded genes, with emphasis on the validated *hsa-miR-26b-5p, hsa-miR-100-5p* and *hsa-miR-143-3p*, as well as *hsa-miR-21-5p* and its high number of target genes. Our findings reinforce some known pathways and suggest novel interactions that might be associated with T1DM and its complications, such as *hsa-miR-26b-5p* and the mitochondrial metabolism.

## Data availability statement

The datasets presented in this study can be found in online repositories. The names of the repository/repositories and accession number(s) can be found below: https://www.ebi.ac.uk/ena, PRJEB51173.

## Ethics statement

The studies involving human participants were reviewed and approved by Institutional Review Board from João de Barros Barreto University Hospital (HUJBB, Belém, Pará, Brazil) (Protocol Number 005/12). The patients/participants provided their written informed consent to participate in this study.

## Author contributions

Conceptualization, ÂR-d-S and JF. Data collection and clinical trial, FTCM, ACCBS, VSGL, PBBF, JFAN, LVM and GNL. Methodology, AV, TV-S and LM. Validation, LS, RC, CB-d-S and LR-d-M. Formal analysis, ÂR-d-S. Investigation, RF and GC. Data curation, ÂR-d-S and AP. Writing-original draft preparation, RF and GC. Writing-review and editing, RF, GC and ÂR-d-S. Visualization, RF and LM. Supervision, LS, NQ and KF. Project administration, ÂR-d-S and JF. Funding acquisition, ÂR-d-S and JF. All authors contributed to the article and approved the submitted version.

## Funding

This study was funded by Rede de Pesquisa em Genômica Populacional Humana (Coordenação de Aperfeiçoamento de Pessoal de Nível Superior - CAPES/Biologia Computacional: No. 3381/2013/CAPES) and Pró-Reitoria de Pesquisa e Pós-Graduação da Universidade Federal do Pará - PROPESP/UFPA. ÂR-d-S was supported by Conselho Nacional de Desenvolvimento Científico e Tecnológico - CNPq/Productivity: 304413/2015-1.

## Acknowledgments

The authors thank the Endocrinology and Metabology/Diabetes Unit of the of the Joao de Barros Barreto University Hospital at the Federal University of Para (HUJBB-UFPA) for providing blood samples. This project was conducted and supported by the Endocrinology and Metabology/Diabetes Unit in collaboration with Human and Laboratory of Human and Medical Genetics.

## Conflict of interest

The authors declare that the research was conducted in the absence of any commercial or financial relationships that could be construed as a potential conflict of interest.

## Publisher’s note

All claims expressed in this article are solely those of the authors and do not necessarily represent those of their affiliated organizations, or those of the publisher, the editors and the reviewers. Any product that may be evaluated in this article, or claim that may be made by its manufacturer, is not guaranteed or endorsed by the publisher.

## Abbreviations

T1DM, Type 1 diabetes mellitus; DM, Diabetes Mellitus; ICA, against islet cells; GADA, glutamate decarboxylase; IAA, insulin; IA-2, tyrosine phosphatase; IA-2β, tyrosine phosphatase; ZnT8, zinc transporter 8; miRNAs, microRNAs; OXPHOS, oxidative phosphorylation; ADA, American Diabetes Association; NGS, Next-Generation Sequencing; DE, Differential Expression; Hg19, Human Genome Reference; RPKM, Reads per Kilobase per Million; RT‐qPCR, Real‐Time Quantitative Reverse Transcription‐PCR; ROC, Receiver Operating Characteristics; AUC, Area Under the Curve; PBMCs, Peripheral Blood Mononuclear Cells; DDR2, Discoiding Domain Receptor 2; ETC, electron transport chain; ROS, reactive oxygen species; GH, growth hormone.
