# Supplementary material for: Global miRNA expression reveals novel nuclear and mitochondrial interactions in Type 1 diabetes mellitus
Source: Front Endocrinol (Lausanne). 2022 Nov 24;13:1033809. doi: 10.3389/fendo.2022.1033809 (PMC9731375; doi:10.3389/fendo.2022.1033809)
Supplement: Supplementary file 1 [file DataSheet_1.docx]

Supplementary Material

Supplementary Figures and Tables


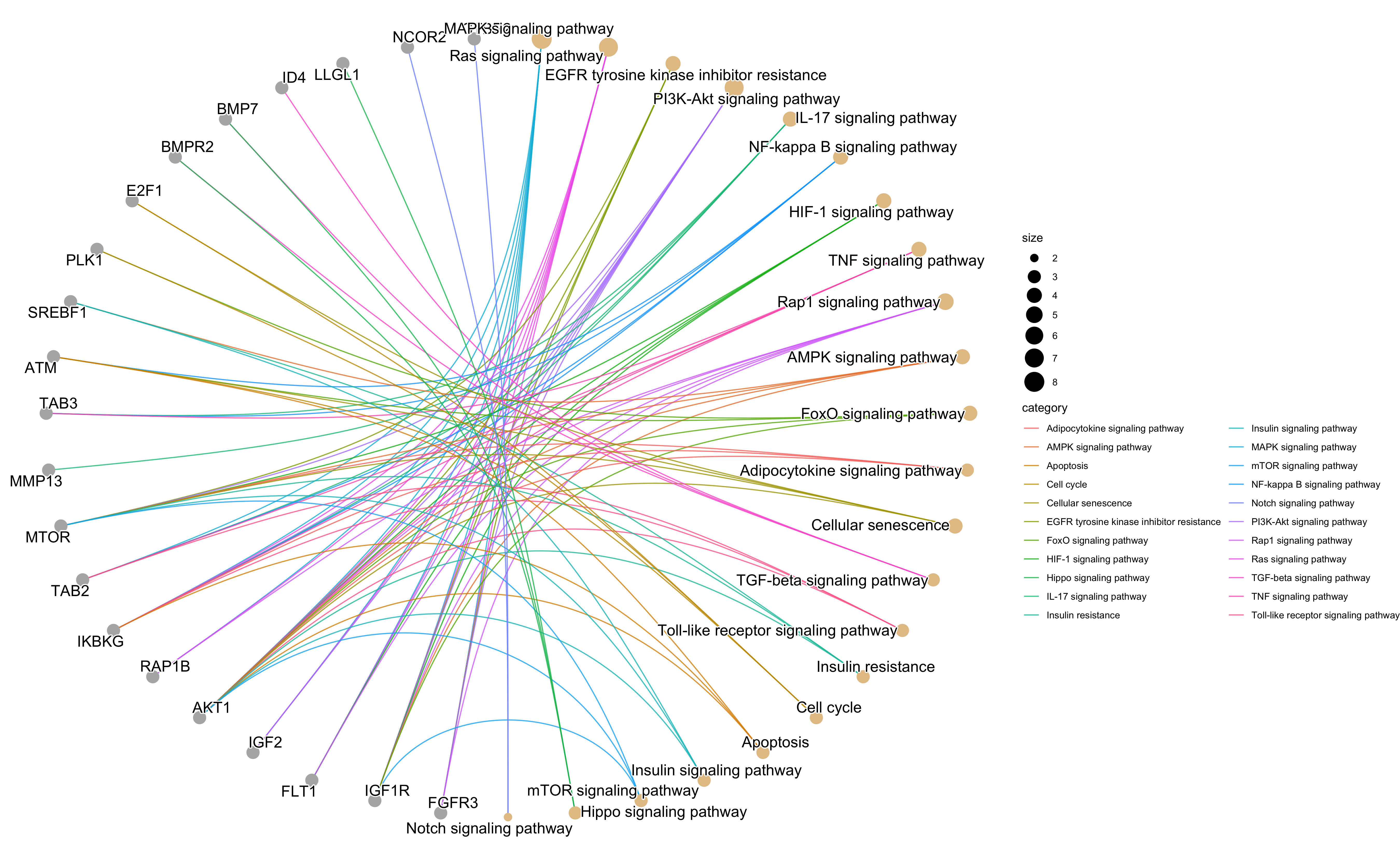


**Supplementary Figure S1** Target genes of the upregulated miRNAs linked to pathways important to the development of T1DM.


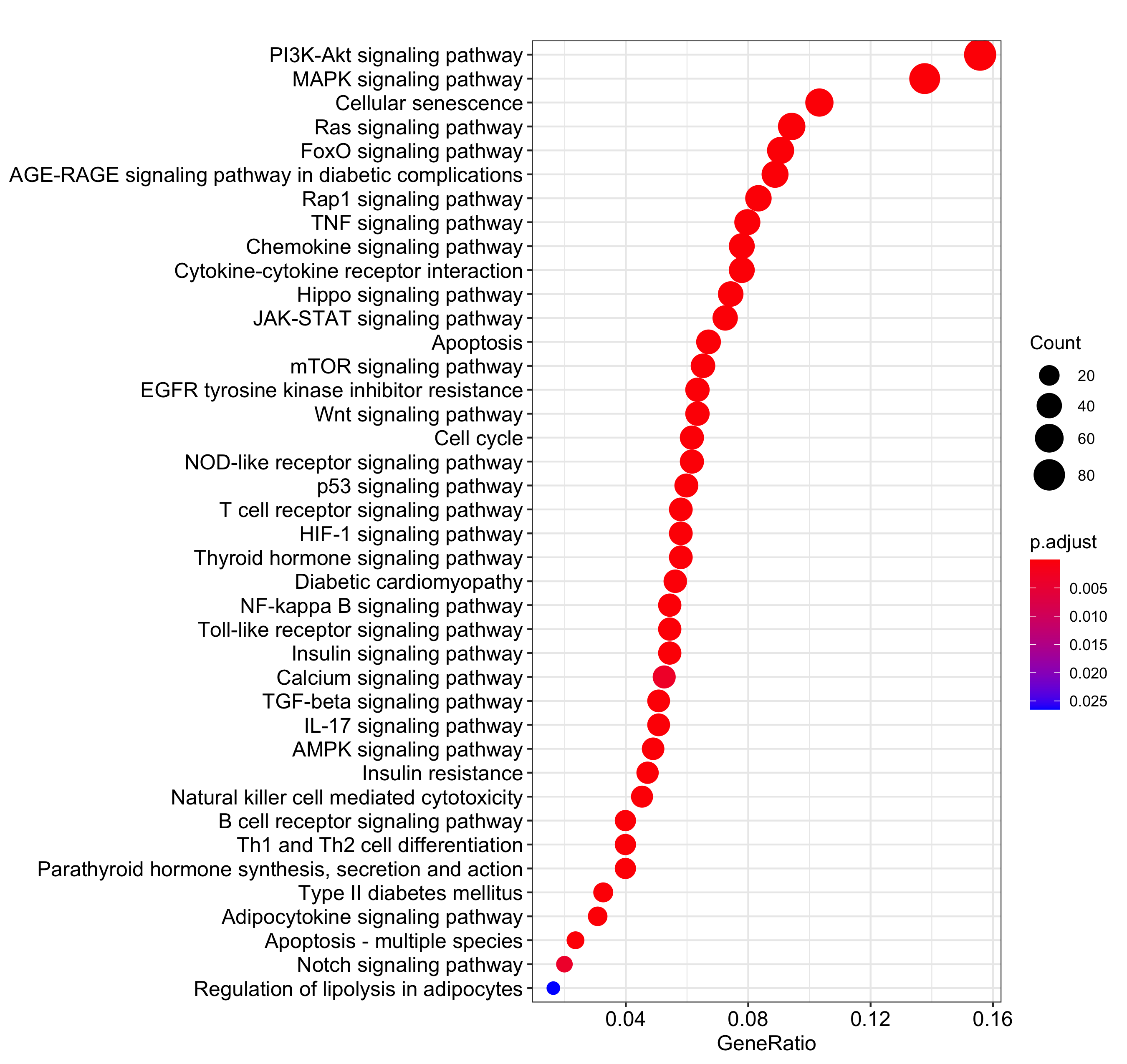


**Supplementary Figure S2** Dotplot showing KEGG pathways enrichment analysis performed to target genes of the downregulated miRNAs. The color of the dot is based on pathways enrichment significance and the dot size on gene count enriched in the pathway.

**Supplementary Figure S3** Analyses of the DE miRNAs that target nuclear genes intrinsically involved in important pathways of the mitochondrial metabolism. **(A)** Network of these miRNAs (in blue or green, the latter being those selected to be validated in the current study) and the mitochondrial genes they target (in yellow); **(B)** Number of miRNAs per mechanism; **(C)** Number of target genes per miRNAs in the investigated mechanisms. *Important pathways to T1DM development.

**Supplementary Figure S4** Enrichment analyses for the miRNAs with nuclear target genes involved in mitochondrial metabolism, considering **(A)** upregulated miRNAs and **(B)** downregulated miRNAs.

**Supplementary Table S1** Specific primers for mature miRNAs and small nucleolar RNA U6.

| Target | Primer forward |
| --- | --- |
| hsa-miR-26b-5p | 5’ TTCAAGTAATTCAGGATAGGT 3’ |
| hsa- let-7i-5p | 5’ TGAGGTAGTAGTTTGTGCTGTT 3’ |
| hsa-miR-143-3p | 5’ TGAGATGAAGCACTGTAGCTC 3’ |
| hsa-miR-501-3p | 5’ AATGCACCCGGGCAAGGATTCT 3’ |
| hsa-miR-100-5p | 5’ AACCCGTAGATCCGAACTTGTG 3’ |
| U6 | 5’ CGCTTCACGAATTTGCGTCAT 3’ |

**Supplementary Table S2** Target genes of differentially expressed miRNAs. These data are part of the article "Global miRNA expression reveals novel nuclear and mitochondrial interactions in Type 1 Diabetes Mellitus". Available in [10.6084/m9.figshare.19576255](https://doi.org/10.6084/m9.figshare.19576255)

**Supplementary Table S3** Functional analysis of the target genes of the upregulated miRNAs. These data are part of the article "Global miRNA expression reveals novel nuclear and mitochondrial interactions in Type 1 Diabetes Mellitus". Available in [10.6084/m9.figshare.19576204](https://doi.org/10.6084/m9.figshare.19576204)

**Supplementary Table S4** Functional analysis of the target genes of the downregulated miRNAs. These data are part of the article "Global miRNA expression reveals novel nuclear and mitochondrial interactions in Type 1 Diabetes Mellitus". Available in [10.6084/m9.figshare.19576198](https://doi.org/10.6084/m9.figshare.19576198)
